# Induction of Lysosomal Membrane Permeabilization Is a Major Event of FTY720-Mediated Non-Apoptotic Cell Death in Human Glioma Cells

**DOI:** 10.3390/cancers12113388

**Published:** 2020-11-16

**Authors:** Kyoung-jin Min, Taeg Kyu Kwon

**Affiliations:** 1 Department of Immunology, School of Medicine, Keimyung University, 1095 Dalgubeoldaero, Dalseo-Gu, Daegu 42601, Korea; kjmin@dgmif.re.kr; 2 New Drug Development Center, Daegu-Gyeongbuk Medical Innovation Foundation, 80 Chembok-ro, Dong-gu, Daegu 41061, Korea; 3 Center for Forensic Pharmaceutical Science, Keimyung University, 1095 Dalgubeoldaero, Dalseo-Gu, Daegu 42601, Korea

**Keywords:** FTY720, non-apoptotic cell death, LMP, cathepsins, glioma

## Abstract

**Simple Summary:**

FTY720, a sphingosine-1-phosphate (S1P) analog, is a potent immunosuppressant for the treatment of multiple sclerosis. In addition to being an immune modulator, FTY720 also features antitumor activity in several cancer models, but the molecular mechanisms are unclear. Here, we extended our research to analyze the signaling pathways mediating FTY720-induced cell death. FTY720 did not induce apoptotic cell death, autophagy, paraptosis, or necroptosis in glioma cells. Interestingly, FTY720 accumulated in lysosomes, resulting in the induction of lysosomal membrane permeabilization (LMP). Inhibition of LMP by overexpression of HSP70 and cathepsin inhibitors blocked FTY720-induced cell death. These data suggest that FTY720 induces cell death induced by LMP in glioma cells.

**Abstract:**

FTY720, a sphingosine-1-phosphate (S1P) receptor modulator, is a synthetic compound produced by the modification of a metabolite from *I. sinclairii*. Here, we found that FTY720 induced non-apoptotic cell death in human glioma cells (U251MG, U87MG, and U118MG). FTY720 (10 µM) dramatically induced cytoplasmic vacuolation in glioma cells. However, FTY720-mediated vacuolation and cell death are not associated with autophagy. Genetic or pharmacological inhibition of autophagy did not inhibit FTY720-induced cell death. Herein, we detected that FTY720-induced cytoplasmic vacuoles were stained with lysotracker red, and FTY720 induced lysosomal membrane permeabilization (LMP). Interestingly, cathepsin inhibitors (E64D and pepstatin A) and ectopic expression of heat shock protein 70 (HSP70), which is an endogenous inhibitor of LMP, markedly inhibited FTY720-induced cell death. Our results demonstrated that FTY720 induced non-apoptotic cell death via the induction of LMP in human glioma cells.

## 1. Introduction

FTY720 is a synthetic compound produced by modification of a metabolite from *I. sinclairii* and has strong anti-cancer activity. For example, FTY720 induces cell death in multiple cancer cells [1,2,3,4] and sensitizes cancer cells to chemotherapy and radiotherapy [5,6,7,8]. Interestingly, FTY720 has also been seen to increase non-apoptotic cell death. For example, FTY720 induces ferroptosis and autophagy in multiple myeloma cells [9], and increases necrotic cell death in ovarian cancer cells [10]. In addition, FTY720 induces caspase-independent cell death in acute lymphoblastic leukemia [11], autophagy-related apoptosis, and necroptosis in human glioblastoma cells [12]. Even though FTY720 induces cell death in a variety of cancer cells, the cell death mode and mechanism by FTY720 in glioma cells are not sufficiently understood.

Lysosomes are acidic organelles for the degradation of intracellular or extracellular macromolecules [13]. Recently, the function of lysosomes has been emphasized in cancer cells. It is well-known that proper fusion between lysosomes and autophagosomes must occur for autophagy flux. The role of autophagy is contradictory in cells, but if autophagy flux does not occur successfully, the viability of cancer cells is affected [14]. In addition, there are many cathepsins, proteases, and other enzymes in lysosomes. These proteins are released into the cytosol via induction of lysosomal membrane permeabilization (LMP) by anti-cancer drugs, and then induce cell death via activation of the lethal process [15,16,17,18,19]. In particular, released cathepsins play a major role in LMP-induced cell death, and inhibitors of cathepsins block LMP-induced cell death [20,21]. LMP has been known to be regulated by levels of heat shock protein 70 (HSP70). Inhibition of HSP70 by 2-phenylethynesulfonamide induces LMP, and released cathepsins induce cancer cell death [22]. HSP70 scavenges lysosomal labile iron to protect lysosomal membranes [23], and stabilizes them, resulting in the inhibition of LMP by diverse stimuli [24,25,26].

Here, we investigated the effect of FTY720 on cell death and the related molecular mechanisms were evaluated in human glioma cells. Our results demonstrated that lysosomal accumulation of FTY720 was induced lysosomal membrane permeabilization, resulted in induction of cell death. By causing cell death by FTY720 separately from existing cell death (apoptosis, necrosis, and autophagy), it will be valuable as a novel anti-cancer drug in cancer treatment.

## 2. Results

### 2.1. FTY720 Increases Cell Death of Glioma Cells in a Caspase-Independent Manner

**Figure 1:**
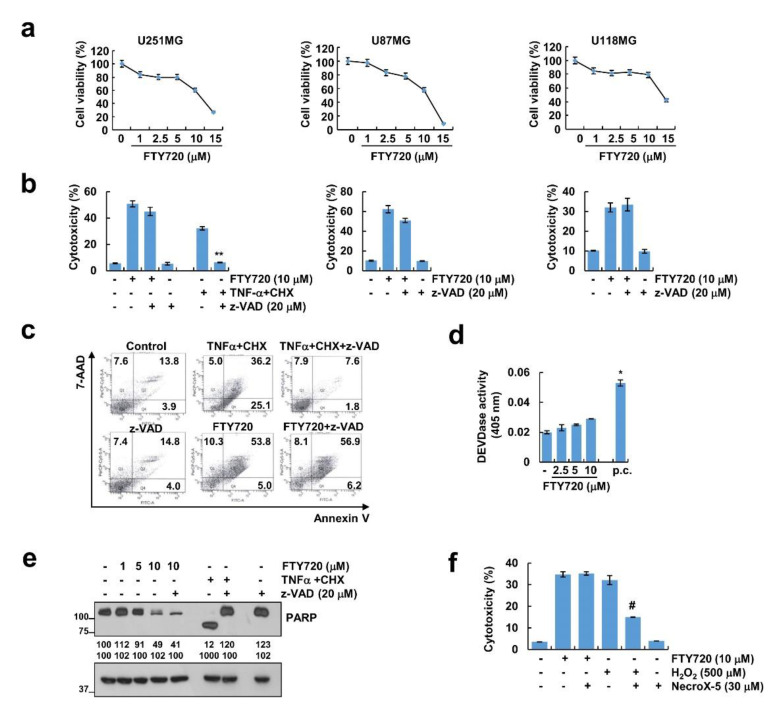
FTY720 induces cell death in human glioma cells. (**a**) Cells (U251MG, U87MG, and U118MG) were treated with the indicated concentrations of FTY720 for 24 h. The cell viability was determined by XTT assay. (**b**) Cells (U251MG, U87MG, and U118MG) were treated with 10 μM FTY720 in the presence or absence of 20 μM z-VAD for 24 h. Cell cytotoxicity was detected by LDH assay. (**c**–**e**) U251MG cells were treated with 10 μM FTY720 or 5 ng/mL TNF-α plus 2.5 μg/mL cycloheximide (CHX) (positive control; p.c.) in the presence or absence of 20 μM z-VAD for 24 h. Cell death was determined by staining with 7-AAD and Annexin V (**c**). Caspase activities were calculated using caspase-3 (DEVDase) assay kits (**d**). Protein expression was detected by Western blotting (**e**). (**f**) U251MG cells were treated with 10 μM FTY720 (18 h) and 250 μM H_2_O_2_ (2 h) in the presence or absence of 20 μM NecroX-5. Cell cytotoxicity was detected by LDH assay. The values in the graphs (**a**,**b**,**d**,**f**) represent the mean ± SD of three independent experiments. ** *p* < 0.01 compared to the TNF-α plus CHX. * *p* < 0.01 compared to the control. ^#^ *p* < 0.01 compared to the H_2_O_2_.

We examined the effect of FTY720 on glioma cell death. We found that FTY720 decreased glioma cell viability in a dose-dependent manner in U251MG, U87MG, and U118MG (Figure 1a). Next, we investigated whether caspase activation is involved in FTY720-induced cell death. Interestingly, although the pan-caspase inhibitor (z-VAD) completely blocked TNF-α plus cycloheximide (CHX)-induced cell death, z-VAD had no effect on cell death in FTY720-treated glioma cells (Figure 1b). To further confirm the caspase-independent cell death induced by FTY720 treatment, we performed flow cytometry analysis with Annexin V/7-AAD double staining [27]. TNF-α plus CHX increased the population of Annexin V(+)/7-AAD(−) and Annexin V(+)/7-AAD(+), but FTY720 only increased the population of Annexin V(+)/7-AAD(+) (Figure 1c). Inhibition of caspase by z-VAD decreased Annexin V(+)/7-AAD(−) and Annexin V(+)/7-AAD(+) populations induced by TNF-α plus CHX (Figure 1c). However, the population of Annexin V(+)/7-AAD(+) induced by FTY720 was not altered by z-VAD treatment (Figure 1c). Furthermore, the activation of caspase and cleavage of PARP could not be measured in FTY720-treated cells (Figure 1d,e). Next, we examined the possibility of necrosis. When cells were treated with NecroX-5, a necrosis inhibitor, cell death by H_2_O_2_ was blocked, but FTY720-induced cell death did not change (Figure 1f). Therefore, these data indicate that FTY720 induces non-apoptotic and non-necrotic cell death in glioma cells.

### 2.2. Inhibition of Autophagy Had No Effect on FTY720-Induced Cell Death in Glioma Cells

**Figure 2:**
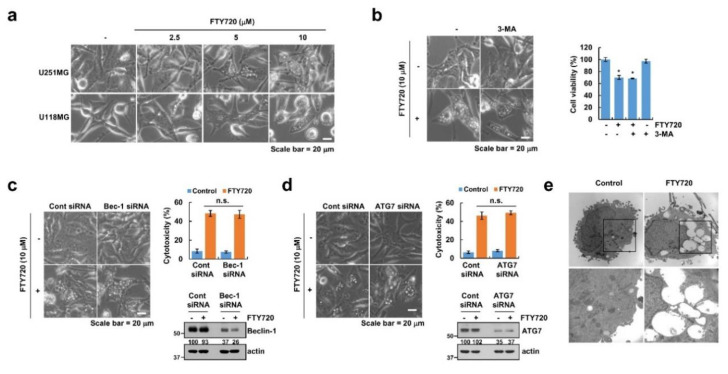
Effect of FTY720 on autophagy in human glioma U251MG cells. (**a**) Cells were treated with the indicated concentrations of FTY720 for 6 h. (**b**) U251MG cells were pretreated with 1 mM 3-MA for 30 min, and then treated with 10 μM FTY720 for 24 h. (**c**,**d**) U251MG cells were transfected with Beclin-1 (Bec-1) siRNA (**c**), ATG7 siRNA, (**d**) and/or control siRNA (Cont siRNA). Twenty-four hours after transfection, cells were treated with 10 μM FTY720 for 24 h. (**e**) U251MG cells were treated with 10 μM FTY720 for 12 h, and cell morphology was detected by electron microscopy. Cell morphology was detected by interference light microscopy. The mean number of microscopic images (**a**–**e**) per sample is 10, and these images were taken randomly. Cell viability was determined by XTT assay (**b**). Protein expression was detected by Western blotting and cell death was analyzed by LDH assay (**c**,**d**). The values in the graphs (**b**–**d**) represent the mean ± SD of three independent experiments. * *p* < 0.01 compared to the control. n.s. = no significance.

As shown in Figure 2a, we found that FTY720 dramatically induced cytoplasmic vacuolation within 6 h. Previous studies reported that vacuolization is related to several types of cell death [28], and cytoplasmic vacuolation is detected in autophagy-related cell death [29]. Therefore, we investigated whether autophagy is critical for FTY720-induced cell death. First, we used 3-methyladenine (3-MA), which inhibits autophagy by disrupting autophagosome formation via inhibition of type III phosphatidylinositol 3 kinase (PI3K). 3-MA did not block cytoplasmic vacuolation or cell death (Figure 2b and Figure S1). In addition, knockdown of autophagy-related genes (*Beclin*-*1* and *ATG7*) by siRNA did not change cell morphology or death (Figure 2c,d). Furthermore, we did not observe the formation of autophagosomes or autolysosomes in the electron microscopy data (Figure 2e). Therefore, these data indicate that autophagy is not associated with FTY720-induced cell death.

### 2.3. FTY720-Induced Cell Death Is Independent of Paraptosis

**Figure 3:**
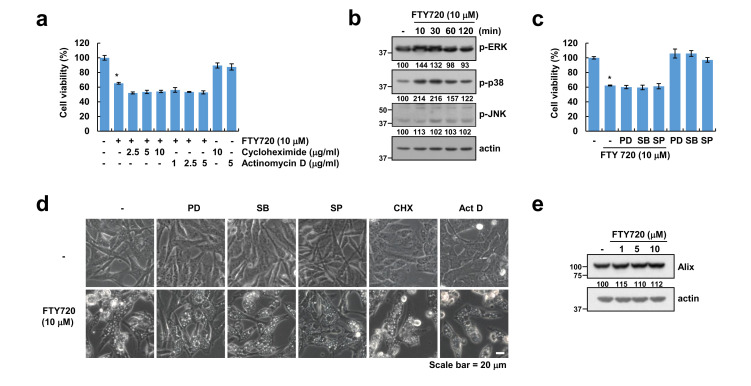
FTY720-induced cell death is independent of paraptosis in human glioma cells. (**a**) U251MG cells were pretreated with the indicated concentrations of cycloheximide or actinomycin D for 30 min, and then treated with 10 μM FTY720 for 24 h. (**b**) U251MG cells were treated with 10 μM FTY720 for the indicated time periods. (**c**,**d**) U251MG cells were pretreated with MAPK inhibitors [ERK inhibitor: 50 μM PD98059 (PD); p38 MAPK inhibitor: 10 μM SB203580 (SB); and JNK inhibitor: 10 μM SP600125 (SP)] for 30 min, and then treated with 10 μM FTY720 for 24 h. (**e**) U251MG cells were treated with the indicated concentrations of FTY720 for 24 h. Cell viability was analyzed by XTT assay (**a**,**c**), and protein expression was detected by Western blotting (**b**,**e**). Cell morphology was detected by interference light microscopy. The mean number of microscopic images per sample is 10, and these images were taken randomly (**d**). The values in the graphs (**a**,**c**) represent the mean ± SD of three independent experiments. * *p* < 0.01 compared to the control.

As paraptosis also induces cytoplasmic vacuolation derived from the endoplasmic reticulum (ER) and mitochondria [30], we investigated the possibility of paraptosis. Seeing that paraptosis is known to be essential for new protein and/or RNA synthesis, both inhibitors can inhibit paraptosis [31]. However, as shown in Figure 3a, neither the de novo protein synthesis inhibitor (cycloheximide) nor the transcription inhibitor (actinomycin D) changed the levels of cell death induced by FTY720 treatment (Figure S2a,b). As it is known that activation of MAPKinase also contributes to paraptosis [32], we checked the activation of MAPKinase. FTY720 slightly increased the phosphorylation of ERK and p38 MAPK (Figure 3b), but inhibitors of MAP kinase [(ERK inhibitor (PD98059), p38 MAP kinase inhibitor (SB203580), and JNK inhibitor (SP600125)] did not impact the levels of FTY720-induced cell death or cytoplasmic vacuolation (Figure 3c,d and Figure S2c). Furthermore, the level of Alix, which is a critical protein that inhibits paraptosis [32], is also unchanged in FTY720-treated cells (Figure 3e). These data suggest that induction of cell death and cytoplasmic vacuolation by FTY720 are independent of paraptosis.

### 2.4. FTY720 Induces Lysosomal Membrane Permeabilization (LMP)

**Figure 4:**
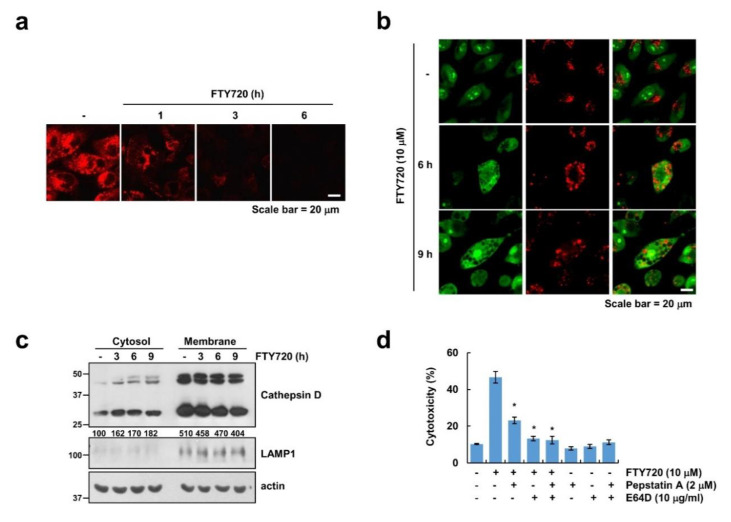
FTY720 induces lysosomal membrane permeabilization (LMP). (**a**–**c**) U251MG cells were treated with 10 μM FTY720 for the indicated time periods. Cells were stained with lysotracker red (**a**) and acridine orange (**b**). Cytosol and membrane fractions (lysosome-rich fraction) were prepared, and the protein levels were determined by Western blotting. LAMP1 is shown as cell compartment controls (lysosome-rich membrane fraction) (**c**). (**d**) U251 cells were pretreated with 2 μM pepstatin A (Pep A) and/or 10 μg/mL E64D for 30 min, and then added along with 10 μM FTY720 for 24 h. Cell toxicity was determined using the LDH assay. The mean number of microscopic images (**a**,**b**) per sample is 10, and these images were taken randomly. The values in the graphs (**d**) represent the mean ± SD of three independent experiments. * *p* < 0.01 compared to the FTY720.

Finally, as recent papers have reported that cell death by LMP also induces cytoplasmic vacuolation [33], we investigated whether FTY720 enhances LMP. To investigate the effect of FTY720 on LMP, we used lysotracker red, an acidotropic probe [21]. FTY720 decreased red fluorescence within 1 h and gradually decreased it until 6 h (Figure 4a). To confirm the FTY720-induced LMP, cells were stained with acridine orange (AO), a lysosomotropic metachromatic fluorochrome. FTY720 decreased the number of red puncta and increased cytosolic acidification, which elevated green fluorescence (Figure 4b). Interestingly, we found that cytoplasmic vacuoles were stained with AO red fluorescence (Figure 4b). Therefore, these data suggested that the induction of cytoplasmic vacuolation by FTY720 might be mediated by lysosome dilation. Interestingly, 9 h after FTY720 treatment, the vacuoles were no longer stained as a red fluorescence by AO, and the number of red puncta was dramatically decreased (Figure 4b). In connection with these results, FTY720 released cathepsin D from the lysosome to the cytosol (Figure 4c). Finally, we investigated whether FTY720-induced LMP is involved in cell death. As LMP-induced cell death is blocked by cathepsin inhibitors [21], we investigated the effect of cathepsin inhibitors on FTY720-induced cell death. Pepstatin A and E64D markedly inhibited cell death in FTY720-treated cells (Figure 4d). Therefore, our data indicated that LMP plays a critical role in FTY720-induced cell death.

### 2.5. Overexpression of Heat Shock Protein 70 (HSP70) Blocked FTY720-Induced Cell Death

**Figure 5:**
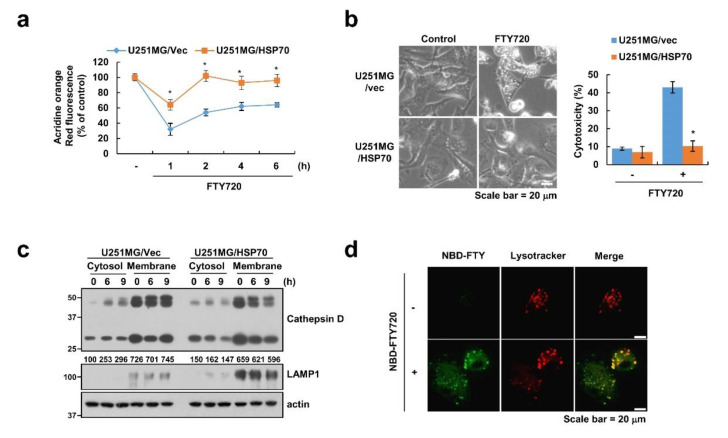
Ectopic expression of HSP70 inhibits FTY720-induced LMP. (**a**–**c**) U251MG/vector and U251MG/HSP70 cells were treated with 10 μM FTY720 for the indicated time periods. Cells were stained with lysotracker red (**a**). Cell morphology was examined using interference light microscopy. Cell cytotoxicity was detected by LDH assay (**b**). Cytosol and membrane fractions (lysosome-rich fraction) were prepared, and the protein levels were determined by Western blotting. LAMP1 is shown as cell compartment controls (lysosome-rich membrane fraction) (**c**). (**d**) U251MG cells were treated with 10 μM NBD-FTY720 for 6 h, and then cell morphology was detected by confocal microscopy. The mean number of microscopic images (**b**,**d**) per sample is 10, and these images were taken randomly. The values in the graphs (**a**,**b**) represent the mean ± SD of three independent experiments. * *p* < 0.01 compared to the FTY720-treated U251MG/vector.

To further confirm LMP-induced cell death, we investigated whether ectopic expression of HSP70 could block FTY720-induced cell death. As shown in Figure 5a, ectopic expression of HSP70 reduced the level and duration of LMP in FTY720-treated cells (Figure 5a). Furthermore, FTY720-induced cytoplasmic vacuolation was not detected, and cell death was inhibited in HSP70-overexpressed U251MG cells (Figure 5b). The release of cathepsins to the cytosol was also decreased by overexpression of HSP70 (Figure 5c). Finally, we examined how FTY720 induces LMP. When we tested the localization of FTY720 after treatment with NBD-FTY720 (fluorescently labeled analog of FTY720), FTY720-NBD was mainly detected in the lysosome (Figure 5d). Therefore, our data suggested that accumulation of FTY720 might induce LMP, resulting in cell death of glioma cells.

### 2.6. Production of Reactive Oxygen Species (ROS) Is Not Involved in FTY720-Induced Cell Death

**Figure 6:**
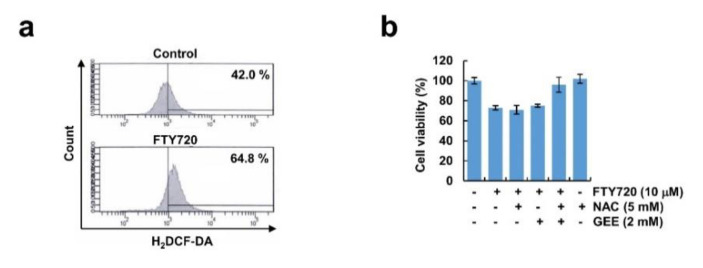
Reactive oxygen species (ROS) had no effect on FTY720-induced cell death in human glioma U251MG cells. (**a**) U251MG cells were treated with 10 μM FTY720 for 3 h, and then cells were stained with H_2_DCF-DA dye. Fluorescence was detected using flow cytometry. (**b**) U251MG cells pretreated with ROS scavengers [5 mM N-acetyl-cysteine (NAC) and 2 mM glutathione ethyl ester (GEE)] for 30 min, and then added along with 10 μM FTY720 for 24 h. Cell viability was determined by XTT assay. The values in the graphs (**b**) represent the mean ± SD of three independent experiments.

Reactive oxygen species (ROS) are important signaling molecules that induce multiple cell death modes, and ROS and LMP can influence each other [34]. Therefore, we tested the involvement of ROS production in FTY720-induced cell death. As shown in Figure 6a, FTY720 increased ROS levels in glioma cells. Even though ROS scavengers [N-acetylcysteine (NAC) and glutathione ethyl ester (GEE)] inhibited ROS generation by FTY720, these scavengers did not block FTY720-induced cell death (Figure 6b and Figure S3). These data suggest that FTY720-induced cell death is independent of ROS production.

## 3. Discussion

The anti-cancer effect of FTY720 is well-known, and the mechanism of cell death by FTY720 has been extensively studied in multiple cancer cells. Here, we suggest a mechanism to induce cell death in glioma. FTY720 induced cell death through apoptosis, autophagy, and a paraptosis-independent manner. FTY720 excessively induced LMP via accumulation of FTY720 in the lysosome. Sequentially, cathepsins were easily released from lysosomes to the cytosol by induction of LMP, and ectopic expression of HSP70 and cathepsin inhibitors markedly inhibited FTY720-induced cell death. Collectively, we found that FTY720 induces LMP-mediated cell death in glioma cells.

FTY720 induces cell death in multiple cancer cells, but the mechanisms of cell death are diverse. First, FTY720 induces apoptosis. It has been reported that FTY720 induces apoptosis in human bladder cancer cells [35], human hepatoma cell lines [36], multiple myeloma cells [37], and pancreatic cancer cells [2]. Second, FTY720 also induces non-apoptotic cell death. Saddoughi et al. reported that FTY720 induces necroptosis [38]. FTY720 activates PP2A through direct binding with I2PP2A/SET oncoprotein, and then induces RIP1K-dependnet necroptosis [38]. In addition, FTY720 induces autophagy, which is involved in cell survival. FTY720 increases non-apoptotic cell death in ovarian cancer cells, and inhibition of autophagy enhances FTY720-induced cell death [10]. In contrast, FTY720-induced autophagy is related to cell death. For example, FTY720 induces autophagy-induced apoptosis in human oral squamous carcinoma cells [39]. FTY720 also increases ferroptosis through activation of PP2A and dephosphorylation of AMPK at Thr172 in multiple myeloma cells [9]. Furthermore, Zhang et al. reported that FTY720 induces autophagy-mediated apoptosis and necroptosis in human glioblastoma cells, including U251MG cells [12]. However, we did not detect apoptosis in U251MG cells (Figure 1). There are some differences between their research and ours. First, they used 15 μM FTY720, which are higher concentrations. When we used 15 μM FTY720, both z-VAD and necrostatin-1 had no effect on FTY720-induced cell death, but combined treatment with pepstatin A and E64D completely blocked 15 μM FTY720-induced cell death (Figure S4a,b). Second, they cells treated with FTY720 for a shorter time than we did. They also suggested that JNK activation is involved in FTY720-induced cell death, as an upstream kinase of autophagy activation [12]. However, SP600125 did not modulate FTY720-induced cell death in our study (Figure 3c). Since JNK activation did not involve in FTY720-induced cell death in our study, we might not detect apoptosis and necroptosis. They suggested that ROS is upstream signaling molecules to induce cell death, but ROS scavengers also had a partial inhibitory effect on FTY720-induced cell death [12]. Thus, in their and our studies, there is the possibility that other death pathways are involved in FTY720-induced cell death in glioma. In our study, inhibitors of Pepstatin A and E64D and overexpression of HSP70 completely blocked FTY720-induced cell death (Figure 4d and Figure 5b). Therefore, we think our study will help researchers to understand the mechanism of FTY720-induced cell death.

Accumulation of FTY720 in lysosomes plays a critical role in glioma cell death. In addition to FTY720, several drugs are accumulated in lysosome. For example, amine-modified polystyrene nanoparticles accumulate in lysosome, and induce LMP, resulting in induction of cell death [40]. In addition, *l*-norephedrine, a sympathomimetic amine, induces intracellular cholesterol accumulation in lysosome, and increases lysosomal vacuolization [41]. Yuan et al. reported that accumulation of cholesterol oxidation products in lysosome induces vacuolization and increases lysosomal damages, which plays a critical role in cell death in macrophage [42]. Interestingly, Ohkuma and Poole examined the effect of weakly basic amines on lysosomal accumulation and vacuolization. They found that all amines that increased vacuolization had lysosomotropic properties, but not all amines with lysosomotropic properties increased vacuolization [43]. Therefore, accumulation of drugs in lysosome is closely related with vacuolization, and it seems that the degree of vacuolization determines whether cell death can be induced or not.

In recent years, there have been many reports about LMP in cancer cells. Normal cells and cancer cells have defense mechanisms to protect against cell death by LMP. The cystatin family is composed of stefins, cystatins, and kininogens, and they are endogenous cysteine cathepsin inhibitors [44]. In addition, HSP70 modulates lysosome membrane integrity. HSP70 depletion and inhibition by 2-phenylethynesulfonamide triggers LMP, resulted in cell death in diverse tumor cell lines and primary effusion lymphoma [22,26]. When we investigated whether combined treatment with HSP70 inhibitors (pifithrin-μ and bufalin) and FTY720 increases cell death, we found that both HSP70 inhibitors slightly increased FTY720-induced cell death in U251 glioma cells (Figure S5). These data suggested that combine treatment with FTY720 and HSP70 inhibitor might have effective anti-cancer activity. In addition, cholesterol accumulation in lysosomes also increases lysosomal membrane stability, and then decreases the release of cathepsins from lysosomes [45]. In spite of the presence of endogenous inhibitors in these cells, if LMP is not suppressed, multiple cell death occurs. Partial LMP, which occurs in only a subpopulation of lysosomes, increases apoptosis [46,47]. In contrast, massive LMP released high concentrations of cathepsins into the cytosol, and then induced necrosis [48,49].

In our study, FTY720-induced LMP was not related to apoptosis, necrosis, or autophagy. Collectively, our results suggest that FTY720 induces LMP-mediated non-apoptotic cell death in human glioma cells. Therefore, FTY720 may be effectively used as an anti-cancer agent in apoptosis-resistant cancer cells. However, further in vivo studies are needed to develop novel therapeutic strategies against cancer cells.

## 4. Materials and Methods

### 4.1. Cells and Materials

Cells were obtained from the American Type Culture Collection (Manassas, VA, USA). All cells were cultured in appropriate medium containing 10% fetal bovine serum (FBS; Welgene, Gyeongsan, Korea), 1% penicillin-streptomycin, and 100 μg/mL gentamycin (Thermo Fisher Scientific, Waltham, MA, USA). All cell lines tested negative for mycoplasma contamination. The lines were authenticated by standard morphologic examination using microscopy. FTY720 was purchased from Echelon Biosciences (Salt Lake City, UT, USA). *N*-acetyl-l-cysteine was obtained from Calbiochem (San Diego, CA, USA). E64D and NBD-FTY720 were purchased from Cayman Chemical (Ann Arbor, MI, USA). TNF-α and z-VAD-fmk were purchased from R&D Systems (Minneapolis, MN, USA). PD98059, SB203580, SP600125, NecroX-5, and pepstatin A were obtained from Enzo Life Science (Farmington, NY, USA). Anti-PARP, anti-Cathepsin D, anti-LAMP1, and anti-Beclin 1 antibodies were purchased from Santa Cruz Biotechnology (Santa Cruz, CA, USA). Anti-phospho-ERK, phosho-p38, anti-phospho-JNK, and anti-Alix were purchased from Cell Signaling Technology (Beverly, MA, USA). The anti-ATG7 antibody was obtained from ProSci Inc. (Poway, CA, USA). The cycloheximide, H_2_O_2_, 3-methyladenine, actinomycin D, glutathione ethyl ester, and anti-actin antibody were obtained from Sigma (St. Louis, MO, USA).

### 4.2. Cell Viability and Cell Toxicity Assay

The XTT assay was employed to measure cell viability using a WelCount Cell Viability Assay Kit (WelGENE, Daegu, Korea). In brief, 24 h after drug treatment, reagent was added to each well and was then measured with a multi-well plate reader (at 450 nm). The release of lactate dehydrogenase (LDH) is an in vitro marker of cellular toxicity. U251MG cells were treated with FTY720 for 24 h, and then the supernatant was used to assay LDH activity, according to the manufacturer’s instructions (Roche Diagnostic Systems, Montclair, NJ, USA).

### 4.3. Annexin V and 7-AAD Staining

FITC-conjugated Annexin V (BD Pharmingen, San Jose, CA, USA) and 7-aminoactinomycin D (7-AAD) (BD Pharmingen) were used for distinguishing cell death mode. Cells were washed twice in cold PBS and resuspended in Annexin V–binding buffer at a concentration of 3 × 10^6^/mL. This suspension (100 μL) was stained with 5 μL of Annexin V-FITC and 5 μL of 7-AAD. 7-AAD is a nucleic acid dye that was used for the exclusion of nonviable cells. The cells were incubated for 15 min at room temperature in the dark. After addition of 400 μL of binding buffer to each tube, cells were analyzed by fluorescence-activated cell sorting (FACS) on a FACS Canto II (BD Biosciences, San Diego, CA, USA).

### 4.4. DEVDase (Caspase-3) Activity Assay

To evaluate DEVDase (Asp-Glu-Val-Asp-ase) activity, cell lysates were prepared after administration of the appropriate treatment. The assays were performed in 96-well plates by incubating 20 μg of cell lysate in 100 μL of reaction buffer (1% NP-40, 20 mM Tris-HCl [pH 7.5], 137 mM NaCl, and 10% glycerol) containing the caspase substrate (Asp-Glu-Val-Asp-chromophore p-nitroanilide; DEVD-pNA) at 5 μM. The lysates were incubated at 37 °C for 2 h. Thereafter, activity was measured at 405 nm using a spectrophotometer.

### 4.5. Western Blotting

Western blotting was performed according to the methods described in our previous study [50]. In brief, the lysates were collected, boiled with 5X sample buffer, and separated by SDS-PAGE. Proteins on the membrane were probed with specific antibodies, and the antibodies were detected by enhanced chemiluminescence (ECL) solution (EMD Millipore, Darmstadt, Germany). Uncropped images for the blots are provided in Figure S6.

### 4.6. Knockdown of Gene by SiRNA

ATG7 and Beclin-1 siRNA were purchased from Santa Cruz Biotechnology (Santa Cruz, CA, USA). The siRNA was transfected into cells using Lipofectamine RNAiMAX (Thermo Fisher Scientific, Waltham, MA, USA).

### 4.7. Measurement of Lysosomal Membrane Permeabilization (LMP)

To monitor lysosomal destabilization, we used LysoTracker Red and acridine orange (AO). Cells were stained with lysotracker red (2.5 μM) or AO (5 μg/mL). Cells were washed twice with phosphate-buffered saline (PBS), and fluorescence was analyzed by confocal microscopy or fluorescence microscopy. Invitrogen (Carlsbad, CA, USA) and Molecular Probes Inc. (Eugene, CA, USA) supplied LysoTracker Red and AO, respectively.

### 4.8. Fractionation of Cytosol and Membrane Extracts

Cells were washed with ice-cold PBS, resuspended in cytosol extraction buffer (250 mM sucrose, 10 mM KCl, 1.5 mM MgCl_2_, 1 mM EDTA, 1 mM EGTA, and 20 mM HEPES) containing 250 μg/mL digitonin and left on ice for 10 min. The lysate was then centrifuged at 13,000× *g* for 90 s. The supernatant (cytosol) was transferred to a new tube, and pellets (membrane fraction) were suspended in lysis buffer. Lysates were centrifuged at 13,000× *g* at 4 °C for 15 min to obtain the supernatant fractions collected as the membrane extract [51].

### 4.9. Measurement of Reactive Oxygen Species

Intracellular accumulation of ROS was determined using the fluorescent probes 2, 7-dichlorodihydrofluorescein diacetate (H_2_DCF-DA). U251MG cells were treated with FTY720. Then, cells were stained with the H_2_DCF-DA fluorescent dye for an additional 10 min. Afterwards, cells were trypsinized and resuspended in PBS, and fluorescence was measured at specific time intervals using a FACS Canto II (BD Biosciences, San Diego, CA, USA).

### 4.10. Statistical Analysis

We repeated the experiments in our studies at least three times, and all data are represented as the means. Statistical analysis was performed by one-way analysis of variance (ANOVA) and post hoc comparisons (Student Newman Keuls) using SPSS (Statistical Package for the Social Sciences, version 22.0) (SPSS Inc.; Chicago, IL, USA). We chose the sample size on the basis of the minimum effects we wished to measure. When *p*-values were <0.05, they were considered significant.

## 5. Conclusions

FTY720 induces LMP via accumulation in lysosomes, resulting in cell death of glioma cells. Anti-cancer strategies employing FTY720 will probably serve as a means to increase the death of cancer cells resistant to apoptosis or necrosis.

## Supplementary Materials

The following are available online at https://www.mdpi.com/2072-6694/12/11/3388/s1, Figure S1: Effect of 3-MA on PP242 plus curcumin-induced apoptosis. Figure S2: Effect of various inhibitors on cancer cells. Figure S3: Effect of NAC and GEE on FTY720-induced ROS generation in U251MG cells. Figure S4. Effect of apoptosis, necroptosis, and autophagy inhibitors on FTY720-induced cell death. Figure S5. Effect of HSP70 inhibitors on FTY720-induced cell death. Figure S6: Uncropped western blot image for Figure 1, Figure 2, Figure 3, Figure 4, Figure 5.
