# Supplementary material for: Induction of Lysosomal Membrane Permeabilization Is a Major Event of FTY720-Mediated Non-Apoptotic Cell Death in Human Glioma Cells
Source: Cancers (Basel). 2020 Nov 16;12(11):3388. doi: 10.3390/cancers12113388 (PMC7696845; doi:10.3390/cancers12113388)
Supplement: Supplementary file 1 [file cancers-12-03388-s001.pdf]

cancers

*Supplementary Materials*

# Induction of Lysosomal Membrane Permeabilization Is a Major Event of FTY720-Mediated Non-Apoptotic Cell Death in Human Glioma Cells

**Kyoung-jin Min and Taeg Kyu Kwon**

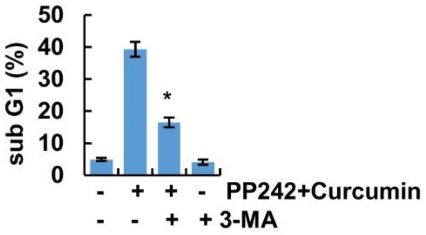


**Figure S1.** Effect of 3-MA on PP242 plus curcumin-induced apoptosis. To verify the effect of 3-MA on cell death, Caki-1 cells were pretreated with the 1 mM 3-MA, and then treated with combined treatment PP242 plus TRAIL for 24 h [1]. Apoptosis was determined by flow cytometry. The values in the graph represent the mean ± SD of three independent experiments. * $p < 0.01$ compared to the combined treatment with PP242 and curcumin.

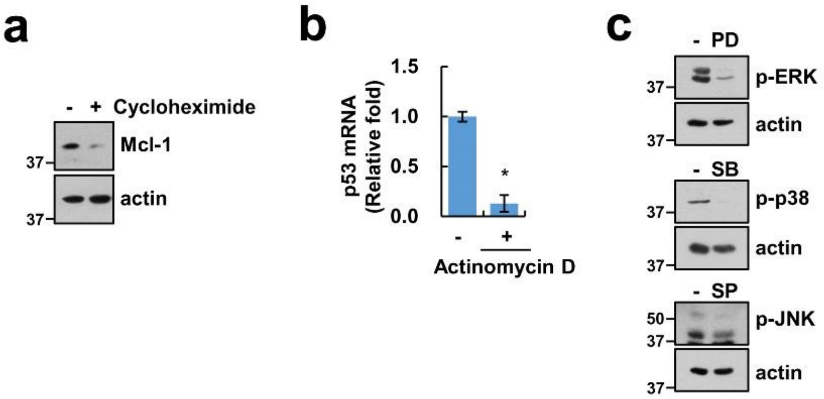


**Figure S2.** Effect of various inhibitors on cancer cells. To verify the effect of various inhibitors [cycloheximide, actinomycin D and MAPK inhibitors (PD98059, SB203580 and SP600125)], U251MG cells were treated inhibitors for 24 h **(a,b)** or 2 h **(c)**. Protein and mRNA expression were determined by western blotting **(a,c)** and qPCR **(b)**, respectively. The values in the graph **(b)** represent the mean ± SD of three independent experiments. * $p < 0.01$ compared to the control.

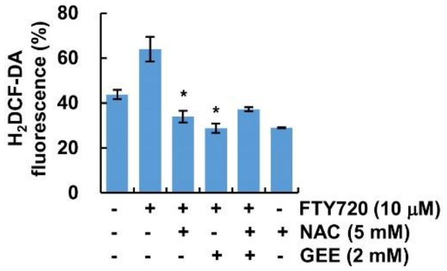


**Figure S3.** Effect of NAC and GEE on FTY720-induced ROS generation in U251MG cells. U251MG cells pretreated with ROS scavengers 5 mM NAC and 2 mM GEE for 30 min, and then added along with 10 μM FTY720 for 3 h, and then cells were stained with H2DCF-DA dye. Fluorescence was detected using flow cytometry. The values in the graph represent the mean ± SD of three independent experiments. * $p < 0.01$ compared to the FTY720.

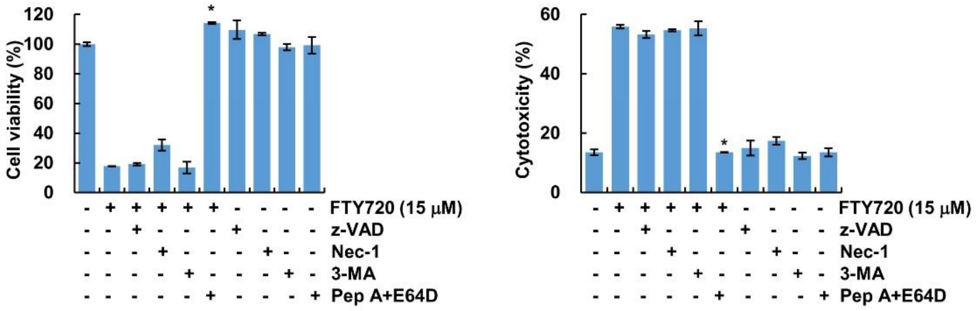


**Figure S4.** Effect of apoptosis, necroptosis, and autophagy inhibitors on FTY720-induced cell death. U251MG cells pretreated with inhibitors (20 μM z-VAD, 60 μM necrostatin-1, and 1 mM 3-MA) and then added along with 15 μM FTY720 for 24 h. Cell viability and cell cytotoxicity were detected by XTT and LDH assay, respectively. The values in the graph represent the mean ± SD of three independent experiments. * $p < 0.01$ compared to the FTY720.

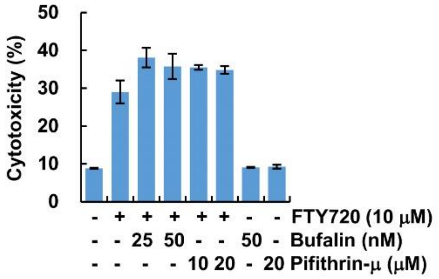


**Figure S5.** Effect of HSP70 inhibitors on FTY720-induced cell death. U251MG cells pretreated with the indicated concentrations of bufalin and pifithrin-μ and then added with 10 μM FTY720 for 24 h. Cell cytotoxicity was detected by LDH assay.

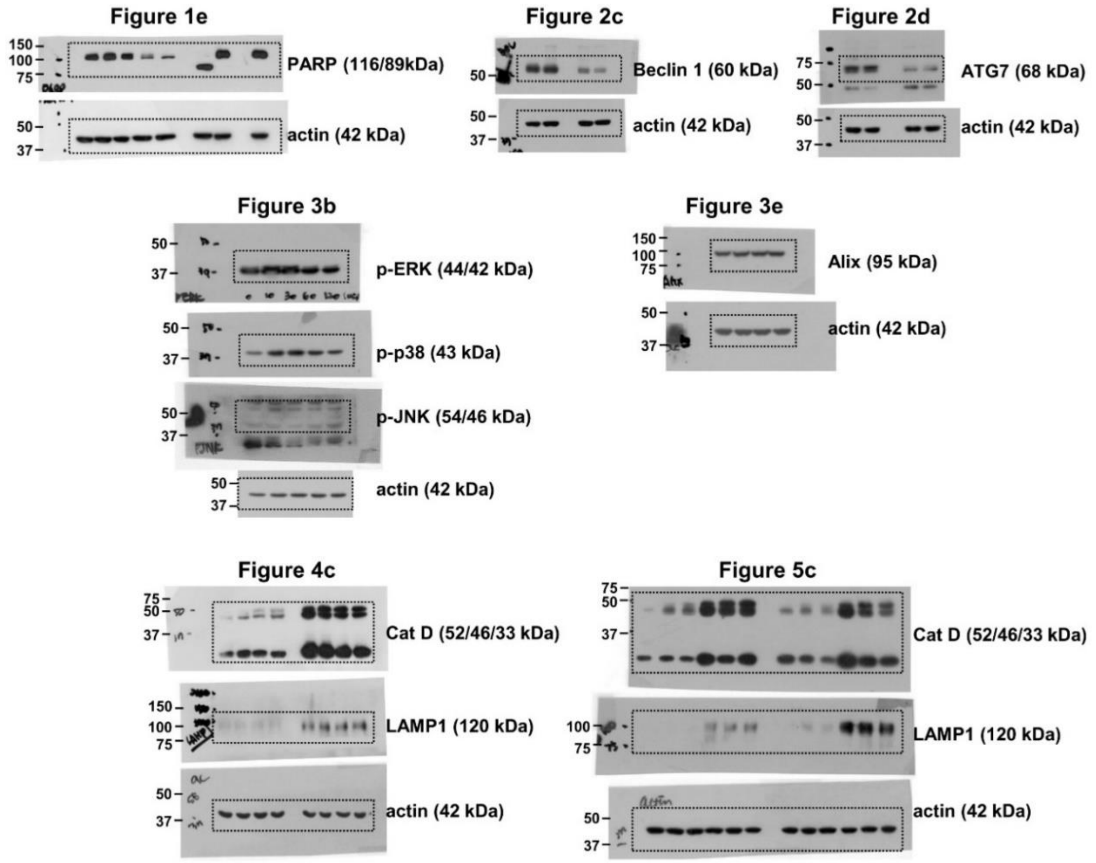


**Figure S6.** Uncropped image of western blot for Figure 1-5.

## References

1. Seo, S. U.; Woo, S. M.; Lee, H. S.; Kim, S. H.; Min, K. J.; Kwon, T. K. mTORC1/2 inhibitor and curcumin induce apoptosis through lysosomal membrane permeabilization-mediated autophagy. *Oncogene* **2018**, *37*, 5205-5220.

© 2020 by the authors. Submitted for possible open access publication under the terms and conditions of the Creative Commons Attribution (CC BY) license (http://creativecommons.org/licenses/by/4.0/).
